# Low Serum Vitamin D in COVID-19 Patients Is Not Related to Inflammatory Markers and Patients’ Outcomes—A Single-Center Experience and a Brief Review of the Literature

**DOI:** 10.3390/nu14101998

**Published:** 2022-05-10

**Authors:** Adina Huțanu, Anca Meda Georgescu, Septimiu Voidăzan, Akos Vince Andrejkovits, Valentina Negrea, Minodora Dobreanu

**Affiliations:** 1 Department of Laboratory Medicine, George Emil Palade University of Medicine, Pharmacy, Science, and Technology of Targu Mures, 540142 Targu Mures, Romania; adina.hutanu@umfst.ro (A.H.); minodora.dobreanu@umfst.ro (M.D.); 2 Department of Laboratory Medicine, Emergency Clinical County Hospital Targu Mures, 540136 Targu Mures, Romania; 3 Department of Infectious Diseases, George Emil Palade University of Medicine, Pharmacy, Science, and Technology of Targu Mures, 540142 Targu Mures, Romania; akos.andrejkovits@umfst.ro (A.V.A.); valentina.negrea@umfst.ro (V.N.); 4 Department of Epidemiology, George Emil Palade University of Medicine, Pharmacy, Science, and Technology of Targu Mures, 540136 Targu Mures, Romania; septimiu.voidazan@umfst.ro; 5 Center for Advanced Medical and Pharmaceutical Research, George Emil Palade University of Medicine, Pharmacy, Science, and Technology of Targu Mures, 540136 Targu Mures, Romania

**Keywords:** COVID-19, inflammation, vitamin D

## Abstract

The aim of the study was to evaluate the vitamin D status in hospitalized COVID-19 patients and the correlation with C reactive protein (CRP), ferritin, fibrinogen, and peripheral blood leukocytes, as well as inflammatory derived indices. A prospective study was performed on 203 COVID-19 hospitalized patients, classified by disease severity. Blood was collected after admission, and inflammatory biomarkers and vitamin D status were assessed using routine laboratory procedures. No significant correlation was found between vitamin D serum levels and disease severity stratified by different age groups. However, the highest vitamin D levels were found in patients with mild disease: median 29.39 (IQR 12.12–44.02) ng/mL, while for moderate and severe forms the serum levels were significantly lower: median 15.10 (IQR 9.56–24.11) ng/mL for moderate, and 18.86 (IQR 12.50–27.88) ng/mL for severe; *p* = 0.009. Patients with no comorbidities showed a significantly higher level of vitamin D median 24.72 (IQR 16.05–31.52) ng/mL compared to subjects with at least one comorbidity: median 16.02 (IQR 9.81–25.22) ng/mL, *p* = 0.004. We did not find an association between vitamin D levels and inflammatory biomarkers except for significantly lower vitamin D levels in moderate and severe COVID-19 compared to mild disease forms.

## 1. Introduction

Vitamin D is a fat-soluble vitamin with an important role in multiple metabolic and immune processes. The body has the capacity to synthesize a large proportion of D3—cholecalciferol when exposed to UVB radiations; it needs further activation by two hydroxylation steps—hepatic and renal [1]. There are many controversies about classifying vitamin D as a hormone. Unlike other vitamins, it is the only one produced by endogenous synthesis from a cholesterol precursor [2]. Vitamin D has multisystemic effects mediated through the vitamin D receptor (VDR) present in various tissues [3], including immune cells [4], and more specifically on leucocytes [5], monocytes [6], and dendritic cells [7]. Activation through T cell receptors (TCRs) induces upregulation of VDR in naïve human T cells and enhances the vitamin D–VDR signaling, important in T cell differentiation and actions. T cells seem to be regulators of VDR expression on innate immune cells modulating the expression of antimicrobial peptides [8]. On B cells, the VDR is constitutively expressed and is also inducible under both active vitamin D and activating signals. Moreover, B cells are able to generate active form 1.25 (OH)_2_ D_3_ from vitamin D precursor by 1 alfa hydroxylase, since the B cells have been found to constitutively express this enzyme [9]. The vitamin D–VDR complex mediates the transcription of at least 1000 genes [10] and also has non-genomic effects such as modulating activities of various enzymes [1]. 

Aside from its well-known beneficial action on bone metabolism, vitamin D is also an important player in the modulation of inflammation and immune response [11]. For the innate immune response, this is achieved by several mechanisms such as (1) modulating the activities of the monocytes/macrophages for both pathogen recognition and phagocytosis [12], (2) a dose-dependent inhibitory effect on macrophage pro-inflammatory cytokines production (IL-6 and TNF alfa) with maximum effect obtained at a concentration of 50 ng/mL vitamin D [13], and (3) induction of the defensins and cathelicidin synthesis, enhancing both the anti-microbial [13], and antiviral defense through the regulation of antimicrobial peptides expression, as reviewed by Ghosh and Weinberg [14]. The effects of antimicrobial peptides on viral infections are related to destabilization of the viral envelope, alteration of the virion structure, and inhibition of the virus binding to the host cell receptors, thus reducing the infectivity [14].

There is evidence that vitamin D reduces the synthesis of pro-inflammatory cytokines interferon (IFN) and interleukin-6 (IL-6) with concomitant reduction in inflammation and the risk of lung lesions due to pneumonia, and increases the synthesis of anti-inflammatory cytokines [15]. In this way, vitamin D seems to play an important role in shifting the immune response from Th1/Th17 toward the Th2 phenotype [15,16,17]. It also stimulates Treg proliferation and increases their functional capacity [18]. Despite the data on the modulatory effects of vitamin D in immune response, in general, and more specifically in SARS-CoV-2, the results are still controversial.

These data convey the assumption that vitamin D might have an important role in enhancing the antimicrobial and antiviral host’s defense. There are studies, as reviewed by Kazemi et al. that analyzed the vitamin D status in patients with COVID-19 [19] and the association between vitamin D levels and the evolution of COVID-19 [20,21,22,23,24], yet the results that emerged are still contradictory. The different outcomes were probably not only due to the type of studies (prospective, retrospective, or case–control), but also due to data collection on the vitamin D supplementation, the dose used, the adjustment for confounders, or by the cut-off definition for the deficiency as a recent meta-analysis revealed. While some studies have established the cut-off at 20 ng/mL, others have set the cut-off point at 10, 12, or 30 ng/mL [19]. Some authors found a close relationship between inflammation markers during SARS-CoV-2 infection and vitamin D status [25], while others did not find a significant difference between the most commonly used inflammatory biomarkers (CRP, ferritin, and IL-6) and vitamin D status [26,27].

Studies on the Romanian population pointed to the high prevalence of vitamin D deficiency [28,29], mostly due to reduced intake in addition to a lower rate of endogenous synthesis, leading to hypovitaminosis D, especially in specific demographic groups (elderly women) and in obese individuals [30]. These specific groups are also prone to more severe forms of SARS-CoV-2 infection [31]. In the current report, the first outcome was the correlation between vitamin D serum levels and disease severity. The secondary outcome was the analysis of the relationship between vitamin D serum level and selected inflammatory biomarkers in a cohort of hospitalized patients from a Romanian tertiary infectious diseases hospital, stratified by disease severity.

## 2. Materials and Methods

### 2.1. Study Design

This was a prospective single-center study conducted between January and September 2021 involving the patients admitted to the 1st Infectious Diseases Clinic Department of Mureș County Clinical Hospital, Târgu Mureș, Romania. The study was conducted in accordance with the Declaration of Helsinki, and the protocol was approved by the Hospital Ethics Committee (No. 19038/21.12.2020). Before initiation of any study procedures and recruitment, the signed informed consent was obtained from the enrolled patients.

Additionally, for comparison purposes, we included a control group derived from the national program for vitamin D testing. For this group (considered here as a control group, COVID-19 negative), we analyzed only the vitamin D serum levels in relation to age and gender; we did not use all personal data, comorbidities, or vitamin D supplementation.

### 2.2. Inclusion Criteria

Adult patients (>18 years old) hospitalized with COVID-19 (Coronavirus Disease 2019) were included. The infection was confirmed with RT-PCR and subsequently, the patients were admitted to the infectious disease department for isolation and treatment; the cohort of patients was recruited between January 2021 and September 2021, when the alfa (B.1.1.7), beta (B.1.351), as well as the delta (B.1.617.2) variant were in circulation [32].

For the control group, the serum vitamin D levels were retrospectively retrieved from the laboratory information system (LIS), including ambulatory care patients between January 2020 and March 2022. The median for each age group was used.

### 2.3. Exclusion Criteria

Patients ≤ 18 years old or diagnosed with possible causes of immunodeficiency (malignancies, HIV infection, hemopathies, or pregnancy) were excluded. Patients unable to read and sign the written informed consent were also excluded. 

### 2.4. Clinical Evaluation for Disease Severity

Patients were classified into three severity categories according to the updated treatment guidelines from the National Institutes of Health (https://www.covid19treatmentguidelines.nih.gov/overview/clinical-spectrum/, 19 October 2021, accessed on 22 March 2022):

1. Mild infection: patients who had any of the various signs and symptoms of COVID-19, but without shortness of breath, dyspnea, or abnormal chest imaging.
2. Moderate infection: patients with evidence of lower respiratory disease during clinical assessment or imaging and with an oxygen saturation (SpO2) ≥ 94% on room air.
3. Severe infection: SpO2 < 94% on room air, a respiratory rate > 30 breaths/min, or lung infiltrates > 50%.

The patients with severe respiratory failure or acute single or multiple organ dysfunctions were transferred to the intensive care unit (ICU).

### 2.5. Sampling Procedure and Data Collection

Demographic data, duration of hospitalization, clinical characteristics (chronic comorbidities, admission to the ICU, survival/clinical outcome, and treatment administrated), and laboratory parameters were routinely collected during the hospital admission. Whole blood collected on EDTA was used for complete blood count (CBC) and analyzed using a fully automated hematological analyzer Sysmex XS-800i (Sysmex Corp, Kobe, Japan). Serum samples collected on clot activator tubes were used for inflammatory serum biomarker evaluation, C-reactive protein (CRP), and ferritin, assessed on Architect 4000c (Abbott Laboratory). Blood samples were collected during the first day after admission; the serum levels of 25-OH vitamin D (total vitamin D) were evaluated from serum aliquots stored at −80 °C.

For 25(OH) vitamin D serum levels, a fully automated assay was used based on a capture electrochemiluminescence (ECLIA) method on a Cobas e411 instrument (Roche Diagnostics). The test allows the quantification of both D3 (25-OH) and D2 (25-OH) vitamins for a total vitamin D estimation. The test protocol uses ruthenium-labeled vitamin D binding protein (VDBP) for analyte capture, and streptavidin-coated microparticles and biotin-labeled 25-OH vitamin D for competition (www.roche.com, accessed on 22 March 2022) [33]. According to the American Endocrine Society, the recommended levels for vitamin D range between 30–50 ng/mL, insufficiency is defined as 21–29 ng/mL, while vitamin D deficiency is defined as a 25(OH) vitamin D ≤ 20 ng/mL [34].

The inflammatory indices were calculated from the routine blood cell count and used in the analyses to fully characterize the relationship between inflammation and vitamin D status in patients with COVID-19. 

Neutrophil to lymphocyte ratio (NLR) was defined as:

NLR = # neutrophils/# lymphocytes, where # denotes the absolute values of neutrophils and lymphocytes

Platelet to lymphocyte ratio (PLR) was calculated as: 

PLR = platelets/# lymphocytes 

Systemic inflammatory index (SII) was also defined as a ratio and expressed as ×10^3^/µL: 

SII = (# neutrophils X platelets)/# lymphocytes 

The SII was identified as a strong predictor for severe disease and invasive ventilation, as well as a worse clinical outcome [35].

### 2.6. Statistical Analysis 

For the statistical analysis, the SPSS software version 22 and Graph Pad 3.6 State Software, San Diego, CA, USA, were used; *p* < 0.05 was considered to be the threshold for significance. For the normal data distribution estimated with the Kolmogorov–Smirnov test, the results were expressed as mean ± SD, while for nonparametric distribution, median and min.–max. values or interquartile range (IQR) were used. The categorical variables were expressed as number (*n*) and percentage (%) of patients from the cohort. A chi-square test was used in order to compare the frequencies of nominal variables. Differences in the mean or median between groups were analyzed using the *t*-test, Mann–Whitney test, and Kruskal–Wallis test with Dunn’s test for multiple comparison groups. Multivariate regression of predictive factors used regression of in-hospital mortality as the dependent variable and demographic parameters, vitamin D status, and inflammatory biomarkers as independent predictive variables in a single model. Results were expressed as hazard ratios (Expβ) with 95% CIs.

## 3. Results

### 3.1. Characterization of the Patients with COVID-19

A total of 203 patients infected with SARS-CoV-2 were analyzed for the vitamin D status in relation to disease severity. The demographic characteristics of the studied population are detailed in Table 1; the age classification was carried out according to the WHO criteria: young age, from 25 to 44 years old; middle age, from 44 to 60 years old; elderly age, between 60 and 75 years old; and senior age, 75 to 90 years old [36]. One very young person, 21 years old, was included in the study and classified accordingly.

The median age of the studied group was 64 (21–87) years, the majority of the patients (89.7%) were older than 45 years, with 58.1% male and 41.9% female. The severe (50.7%) and moderate (42.4%) cases were more frequent than the mild (6.9%) forms of COVID-19. The majority of patients (84.3%) presented at least one comorbidity; the most prevalent was hypertension (62.6%).

### 3.2. Comparative Analysis of the Vitamin D Serum Levels for COVID-19 Patients and the Control Group

The retrospective analysis of vitamin D serum levels in outpatients, addressed through the national vitamin D program, from the laboratory information system (LIS) was performed. After the exclusion of the outliers, a very high number of results remained (*n* = 1830), which is nine times higher than the patients’ group. Out of the total of 1830 subjects, 1593 (87%) were women and only 237 (13%) were male. The median vitamin D serum level for the control group was slightly lower 27.12 (IQR 21.14–36.62) ng/mL for women compared to 29.08 (IQR 21.81–35.89) ng/mL for males, without reaching the statistical significance (*p* = 0.678). The median value for the overall control group was 28.13 (IQR 21.22–36.61) ng/mL, a value close to the vitamin D levels in mild COVID-19 patients.

After the analysis of the vitamin D serum levels in relation to age in the control group, the corresponding vitamin D values were identified: for the 21–44 year age group, a median of 24.90 (IQR 19.62–31.71) ng/mL; for the 45–60 group, median 28.65 (IQR 21.53–36.78); for the 61–75 group, median 30.96 (IQR 22.61–39.71) ng/mL; for the 76–90 group, a median of 30.26 (IQR 20.76–39.08) ng/mL was found; *p* = 0.001 when comparing the extreme groups of youngest and oldest patients and also *p* = 0.0001 for multiple group comparisons.

The comparative analysis of vitamin D serum levels between COVID-19 patients and non-COVID patients is detailed in Table 2. Except for the age group 21–44 years, there was a significant difference in vitamin D serum levels, with higher values in non-COVID-19 subjects compared to COVID-19 patients. However, due to the difference between the number of subjects for the two compared groups (with and without COVID-19), the results must be interpreted with caution. Additionally, while in the studied COVID-19 patients, there was a relative balance regarding the gender (58.1% male and 41.9% female), among the subjects included in the control group (COVID-19 negative) for the retrospective analysis of the vitamin D levels, women were prevalent (87%).

### 3.3. Characterization of the COVID-19 Patients in Relation to Vitamin D Status and Inflammatory Biomarkers 

In our study, vitamin D deficiency was observed in 118 patients (57.20%) with a median of 11.85 (IQR 8.95–16.38) ng/mL; it was more prevalent in significantly older patients. 

The main demographic, clinical and biological characteristics of the COVID-19 patients after dichotomization of the study population according to the vitamin D status (vitamin D deficiency ≤ 20 ng/mL) are presented in detail in Table 2. The median time for hospitalization was 13 days (min. 1–max. 36 days), *n* = 39 (18.75%) patients were admitted or transferred to ICU, and *n* = 25 (12.3%) of the subjects died during hospitalization. Except for age and heart rate, there was no difference between the two groups (Table 3).

The median vitamin D levels for women were significantly lower than in the male population: 14.75 (IQR 10.25–25.56) ng/mL vs. 19.59 (IQR 11.51–27.52) ng/mL, *p* = 0.04.

After Spearman correlation analysis, we did not find a significant relationship between vitamin D serum levels and disease severity stratified by four different age groups, possibly due to the relatively small sample size/group.

**Figure 1:**
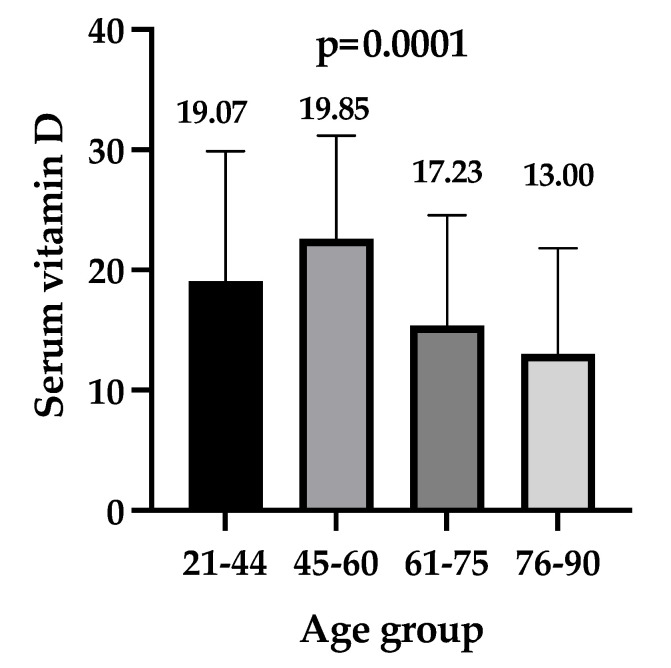
Serum levels of vitamin D were stratified by age groups. Results were analyzed with the Kruskal–Wallis test and Dunn’s for multiple comparisons groups. Box plot (median and interquartile range): the horizontal lines depict the 75% percentile, and the top of the box plot is the median.

After the analysis of the vitamin D serum levels in relation to age, the smallest values were identified at the extreme ages (76–90 years) with a median of 13.0 (IQR 8.83–21.55) ng/mL, while for the 21–44 age group, the median value of vitamin D was 19.07 (IQR 16.77–29.51) ng/mL, *p* = 0.005 when comparing the extreme groups of youngest and oldest patients and *p* = 0.0001 for multiple group comparisons (Figure 1). 

**Figure 2:**
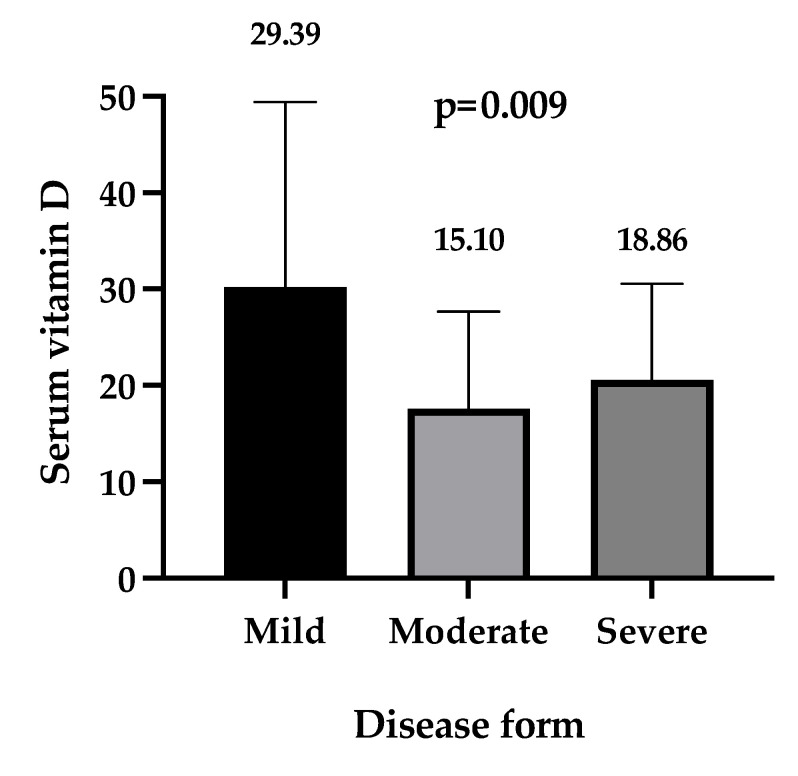
Serum levels of vitamin D in different disease severity. Results were analyzed with the Kruskal–Wallis test and Dunn’s for multiple comparisons groups. Box plot (median and interquartile range), the horizontal lines represent the 75% percentile, and the top of the box plot is the median.

The analysis of the vitamin D status in relation to disease severity showed the highest value in mild COVID-19 patients, with a median of 29.39 (IQR 12.12–44.02) ng/mL, while for moderate and severe forms, the serum levels were significantly lower, with a median of 15.10 (IQR 9.56–24.11) ng/mL for moderate forms, and 18.86 (IQR 12.50–27.88) ng/mL for severe forms, and *p* = 0.009 for all cases (Figure 2). The highest prevalence of moderate and severe COVID-19 forms was found among vitamin D deficient patients.

When vitamin D level was analyzed in relation to comorbidities, the patients with no personal history of illness showed a significantly higher level of vitamin D, with a median of 24.72 (IQR 16.05–31.52) ng/mL compared to subjects with at least one comorbidity, with a median of 16.02 (IQR 9.81–25.22) ng/mL, *p* = 0.004. 

A significant difference in serum levels of vitamin D was found in hypertensive patients, with a median of 16.0 (IQR 9.85–25.28) ng/mL compared to those without hypertension, with a median of 19.71 (IQR 12.88–28.41) ng/mL, *p* = 0.01. Similarly, lower values were found in patients with diabetes mellitus, with a median of 11.03 (IQR 7.45–20.66) ng/mL compared to patients without diabetes mellitus, where the median was 18.75 (IQR 11.78–27.60) ng/mL, *p* = 0.008. For other comorbidities, we did not find a statistically significant difference in vitamin D serum levels (*p* > 0.05). 

The univariate analysis among survivors (*n* = 178) and non-survivors (*n* = 25) is detailed in Table 4. Except for the inflammatory biomarkers, the vitamin D status was not significantly different between groups. 

None of the analyzed laboratory inflammatory parameters showed significant differences between the groups with and without vitamin D deficiency (*p* > 0.05). Demographic and laboratory parameters (age, leucocytes, NLR, PLR, SII, ferritin, and leucocytes) were each significantly related to in-hospital mortality while vitamin D serum levels were not (Table 5).

## 4. Discussion

In this study, we assessed the status of vitamin D in patients infected with SARS-CoV-2. Our patient cohort was graded as mild to moderate/severe COVID-19 based on clinical findings, imagistic parameters and SpO_2_ on room air, the necessity for mechanical ventilation, and the presence of pulmonary infiltration. The extensive pulmonary and multiorgan lesions after the activation of the cytokine storm ultimately lead to acute respiratory distress syndrome and multiorgan failure, respectively. There are several studies that underline the effect of vitamin D in modulating the inflammatory response. Interestingly, a recent paper by Kongsbak-Wismann et al. showed a normal T cell and B cell response during a SARS-CoV-2 infection in one homozygous and two heterozygous patients for mutations in VDR. These mutations totally abolish transcriptional activity after the vitamin D binds the receptor [37]. In the above-mentioned patients, the specific T cell response characterized by IFN gamma, IL-2, and TNF secretion as well as B cell response characterized by anti-RBD IgG antibodies were preserved [37]. This demonstrates that an efficient immune response during SARS-CoV-2 infection does not require vitamin D signaling; however, vitamin D may influence the course of the disease by modulating other antiviral mechanisms, such as antibacterial peptides [14]. An experimental study on vitamin D supplementation in mice with *Pneumocystis murina* pneumonia revealed its anti-inflammatory actions due to the cytokine synthesis modulation, increased expression of toll-like receptors (TLRs) 2 and 4, and glutathione reductase, thus reducing the oxidative stress [13].

The reduction in the vitamin D levels with age and the seasonal variation, with the lowest values during the cold season, seems to be in relation to higher case-fatality rates in older patients [15] and similarly with an increased seasonal incidence of the viral pathology. In a recent study, Dror et al. found that hypovitaminosis D before SARS-CoV-2 infection was associated with more severe/critical illness, compared to patients with moderate/mild form, and the mortality rate was higher among patients with vitamin D insufficiency [38]. Similar to the previously mentioned study, we found that vitamin D deficiency is prevalent in moderate/severe disease forms and advanced age, compared to mild disease forms and younger subjects. A recent article published by Gallelli et al. on a group of 117 individuals studied with and without COVID-19 revealed a significant difference in vitamin D status between groups, with lower levels found in acute COVID-19 compared to non-COVID-19 patients. Similarly, when we analyzed the vitamin D status in COVID-19 patients versus the control group for the same age categories, we found a significant difference, with lower values in infected patients compared to controls (*p* < 0.0001), except for the age group 20–44 years (*p* > 0.05). Additionally, in patients with severe hypovitaminosis D, Gallelli et al. found that IL-6 serum levels were not significantly increased; however, the supplementation with active vitamin D improved the clinical status and IL-6 serum levels in acute disease [39]. Another study found a significant relationship between low levels of vitamin D and increased inflammatory biomarkers, as well as the disease severity [20]. A report by Jain et al. revealed that inflammatory biomarkers are significantly increased in the hypovitaminosis D group and associated with a higher fatality rate [25]. Although we did not find a significant correlation between inflammatory biomarkers and vitamin D levels, the proportion of transfers to ICU was higher among vitamin D deficient patients. 

De Smet et al. found a strong association between low vitamin D levels on admission and mortality; however, the study included only patients with severe respiratory syndrome and the endpoint of the survival rate [40]. In our study, half of the patients were with mild/moderate forms of COVID-19, which might be a reason for the lack of predictive capacity for vitamin D levels related to patients’ outcomes. In a retrospective case–control study, authors found lower levels of vitamin D in hospitalized patients compared to the control group; however, when considering the COVID-19 group, there was no correlation between vitamin D levels and disease severity [26]. In the same study, no other inflammatory biomarkers were found to be significantly different between patients with and without vitamin D deficiency [26].

The supplementation strategy and the frequency of serum vitamin D level determination for both COVID-19 prevention and severity were extensively reviewed by Grant et al. These authors stated that most randomized controlled trials have not reported a significantly reduced risk of infection after vitamin D supplementation [15].

Since there are some reports on the lack of relation between low levels of vitamin D and inflammation biomarkers, it is possible that the mechanisms for positive effects on viral infections are modulated by means of antibacterial peptides, cytokine, and chemokine response in immune cells exposed to the pathogens, or by the effects on viral cell entry or viral replication, and modulatory effect on IFN signaling pathway. Indeed, a recent review highlighted the important role of vitamin D in the innate antiviral immune response [41]. There is increasing evidence that calcitriol is involved in the production of antimicrobial peptides (AMP), enhancement of TLR signaling, and more importantly, the induction of LL-37 synthesis [41]. The review underlined the LL-37 in antiviral defense by multiple mechanisms, one of the important ones being the blockade of the interaction between spike SARS-CoV-2 protein and ACE2 receptor, thus blocking the viral entry. Additionally, LL-37 facilitates the recognition of the nucleic viral material by the endosomal TLRs and activates the IFN response through TRL3, TLR7, and TLR9 signaling [41]. Moreover, human beta-defensin-2 (HBD-2) acts as a calcitriol-induced antiviral protein with a potential protective role in SARS-CoV-2 infection [41]. 

In addition to activation of vitamin D by renal alfa 1 hydroxylase, the active vitamin D forms could be produced under the collective action of VDR (considered a ligand-activated transcription factor) and CYP27B1 extensively expressed on various cells, including macrophages and dendritic cells. This extrarenal production of active vitamin D is regulated by cytokine Th1 signaling (IFN gamma) and TLR1 and TLR2 [42]. A sufficient level of vitamin D is required for a normal IFN gamma-induced antimicrobial peptide expression in macrophages [43], yet a dysregulated or delayed IFN gamma response will allow viral immune evasion [44] and severe forms of COVID-19 after IFN gamma upregulation during the later phase with enhanced cytokine production and hyper-inflammation, thus leading to severe disease [45]. However, a randomized clinical trial evaluating the effect of a single high dose of vitamin D3 in patients with COVID-19, found no significant difference for the in-hospital mortality between group and placebo, although after a single dose, the vitamin D levels increased significantly from 19.8 ng/mL to 44.4 ng/mL [46].

A study by Mardani et al. revealed lower levels of vitamin D in positive SARS-CoV-2 patients compared to the negative group, and that patients with lower vitamin D levels exhibit a higher expression of angiotensin-converting enzyme (ACE) [47], thus interfering with the rennin–angiotensin–aldosterone system RAAS and proper functionality of the immune system through VDR. It is worth mentioning that the synthesis of active vitamin D is maintained even at low/deficient substrate (precursor) levels for up to several months [48]. 

Our results are similar to other recent publications revealing a lack of association between vitamin D levels and severity, or disease outcome [49,50]. On the other hand, in a systematic review of 54 studies regarding the outcome of SARS-CoV-2 infection and vitamin D status, the authors found a strong relationship between low vitamin D levels and admission to ICU and mortality due to COVID-19, or susceptibility to SARS-CoV-2 infection [21]. However, a large vitamin D Mendelian randomization study assessed the evidence for causality between vitamin D levels and severity and susceptibility for SARS-CoV-2 infection; after all the potential confounders were excluded, no association was found [51]. Another large study conducted on more than 300,000 participants failed to prove the beneficial role of vitamin D in protection against severe disease or mortality due to SARS-CoV-2 infection, after adjustment for confounders [52]. Another large-scale Mendelian randomization study for the identification of many traits for severe COVID-19 was performed by Sun et al. using a COVID-19 genome-wide association study (GWAS): host genetics initiative (HGI) for comparison between severe COVID-19 patients with respiratory failure and controls, a HGI study for hospitalized COVID-19 patients with control comparison, and a NEJM study that compared COVID-19 patients with controls in Spain and Italy [53]. Among studied risk factors, the authors analyzed the genetically predicted vitamin D traits (ukb-d-30890_raw) and found neither association with the risk of COVID-19 severity by comparing COVID-19 patients with confirmed severe respiratory symptoms to a control population, nor for hospitalized COVID-19 patients compared to the population control, and for COVID-19 patients with respiratory failure compared to healthy controls from Italy and Spain [53]. 

Mendelian randomization allows the creation of a study design that is not susceptible to environmental or confounding factors, an important aspect especially in studies for vitamin D. In this regard, genetically predicted vitamin D analysis is a more promising parameter for drawing pertinent conclusions. Another Mendelian randomization study performed by Liu et al. did not find evidence supporting genetically predicted vitamin D concentration to be significantly associated with COVID-19 severity or disease susceptibility [54].

Our study has several limitations. First, the serum level of interleukin-6, a potent pro-inflammatory cytokine, was not assessed. Second, the number of the included subjects was relatively low, with a potential impact on results; hence, an additional study would be useful for clarification of the vitamin D involvement in infections in general, and especially in COVID-19 patients. Another potential limitation is the unbalanced proportion between the healthy control group (vitamin D retrieved from a retrospective analysis) and COVID-19 patients. Nevertheless, although few studies have been performed to assess the prevalence of vitamin D deficiency in the Romanian population, they all indicate a high prevalence of vitamin D deficiency, especially in extreme age groups and women [28]. In our study, retrospective analysis of vitamin D serum levels categorized by age in subjects from outpatient settings (otherwise not fully analyzed, unpublished data) revealed that except for the age interval 21–44 years where we found vitamin D insufficiency, in the rest of the age groups, the medians of the vitamin D serum levels were at the lower threshold of the recommended interval, around 30 ng/mL. The vitamin D supplementation before the study adherence was not assessed as a part of the study, only the baseline vitamin D status was assessed shortly after hospital admission.

## 5. Conclusions

Except for significantly lower vitamin D levels in moderate and severe COVID-19 forms compared to mild disease forms, we did not find an association between vitamin D levels and patients’ outcomes or with the inflammatory biomarkers. Compared to the non-COVID-19 group, SARS-COV-2 infected patients had lower vitamin D serum levels, except for the age group 21–44 years, where the difference was without significance. Future studies on a larger cohort, which also include an appropriate control group, are needed for a better characterization of the relationship between vitamin D status and viral infections, in general, and SARS-CoV-2 infection, in particular.

## Figures and Tables

**Table 1:** Distribution of patients in relation to demographic data and comorbidities.

Age (Years)	*n* = 203 (%)
Mean (SD)	63.7 (14.2)
Age group	
21–44 years	21 (10.3%)
45–60 years	60 (29.6%)
61–75 years	70 (34.5%)
76–90 years	52 (25.6%)
Gender *n* (%)	
Male	118 (58.1%)
Female	85 (41.9%)
Disease severity among cases *n* (%)	
Mild	14 (6.9%)
Moderate	86 (42.4%)
Severe	103 (50.7%)
ICU	39 (18.75%)
Death	25 (12.3%)
Comorbidities *n* (%)	
Without comorbidities	32 (15.7%)
Hypertension	127 (62.6%)
Diabetes mellitus (DM)	35 (16.8%)
Dyslipidemia	64 (31.5%)
Obesity	76 (37.4%)
Asthma	7 (3.4%)
COPD	6 (3.0%)
Chronic renal disease	16 (7.9%)
Chronic hepatopathy	2 (1.0%)

Results are expressed as number and percent. ICU = intensive care unit, COPD = chronic obstructive pulmonary disease.

**Table 2:** The comparative analysis of vitamin D serum levels between COVID-19 and non-COVID-19 patients.

Age Groups	Control Group (*n* = 1830)	Patients Group (*n* = 203)	*p*-Value
21–44 years	24.90 (19.62–31.71)	19.07 (16.77–29.51)	0.110
45–60 years	28.65 (21.53–36.78)	22.59 (14.81–31.22)	<0.0001
61–75 years	30.96 (22.61–39.71)	15.37 (9.89–24.22)	<0.0001
76–90 years	30.26 (20.76–39.08)	13.00 (8.83–21.55)	<0.0001

Values are expressed in ng/mL, as median and IQR, after analysis with the Mann–Whitney test for independent samples.

**Table 3:** The demographic and clinical data after patient dichotomization according to vitamin D deficit.

Parameters	Vit D ≤ 20.0 ng/mL *n* = 118	Vit D > 20.0 ng/mL *n* = 85	*p* Value
Baseline characteristics			
Age (years), mean (SD)	65.8 ( ±14.3)	60.79 (±13.6)	0.01 *
Male, *n* (%)	62 (52.5)	56 (65.9)	0.057 **
Hypertension, *n* (%)	79 (66.9)	48 (56.5)	0.13 **
Diabetes, *n* (%)	17 (14.4)	7 (8.2)	0.18 **
Cardiovascular Diseases *n* (%)	26 (22.0)	19 (22.4)	0.95 **
COPD, *n* (%)	5 (4.2)	1 (1.2)	0.20 **
Clinical data			
O2 Saturation, mean (SD)	91.89 (5.44)	90.86 (6.12)	0.22 *
Ventricular allure, mean (SD)	84.34 (15.9)	89.58 (14.9)	0.018 *
Disease form			
Mild, *n* (%)	6 (5.1)	8 (9.4)	0.16 **
Moderate, *n* (%)	56 (47.5)	30 (35.3)	
Severe, *n* (%)	56 (47.5)	47 (55.3)	
Disease outcome			
ICU admission, *n* (%)	21 (17.8)	15 (17.6)	0.97 **
Death, *n* (%)	15 (12.7)	10 (11.8)	0.84 **
Length of hospitalization (days), median (IQR)	13 (10–16)	13 (10.5–15.2)	0.83 ***
Laboratory parameters			
Vitamin D ng/mL, median (IQR)	11.85 (8.95–16.38)	28.20 (24.10–34.80)	<0.0001 ***
Leucocytes ×10^3^/µL, median (min.–max.)	6975.5 (1462–46,100)	7931 (2434–54,600)	0.15 ***
Lymphocytes ×10^3^/µL, median (min.–max.)	1225 (200–9967)	1300 (380–9674)	0.43 ***
Neutrophils ×10^3^/µL, median (min.–max.)	5227 (3597.7–7842.2)	5150 (1080–51,000)	0.53 ***
NLR	4.32 (0.28–55.15)	3.88 (0.34–72.70)	0.90 ***
PLR	156.7 (16.37–876.50)	176.9 (11.20–683.70)	0.62 ***
SII median (min.–max.)	675.63 (36.74–96,677.79)	868.00 (40.91–8716.36)	0.35 ***
CRP (mg/L), median (min.–max.)	43.06 (0.12–254.0)	33.09 (0.12–320.0)	0.47 ***
Ferritin (ng/mL), median (min.–max.)	605.0 (90.0–3647.9)	656.0 (95–4555.6)	0.57 ***
Fibrinogen (mg/dL), median (min.–max.)	446.5 (102.0–900.0)	487.0 (128.7–900.0)	0.56 ***

* Student test, ** chi-square test, *** Mann–Whitney test. ICU = intensive care unit; COPD = chronic obstructive pulmonary disease; NLR = neutrophile to lymphocyte ratio; SII = systemic inflammatory index; PLR = platelet to lymphocyte ratio; CRP = C-reactive protein.

**Table 4:** The characteristics of the surviving vs. non-surviving patients as related to the inflammatory parameters and vitamin D status.

Parameter	Survivors *n* = 178	Non-Survivors *n* = 25	*p*-Value
Age mean (SD)	63.0 (14.4)	70 (10.6)	0.026 *
Male, *n* (%)	106 (89.8%)	12 (10.2%)	0.06 **
Laboratory parameters			
Vitamin D ng/mL, median (IQR)	17.85 (9.87–28.08)	17.08 (11.19–26.58)	0.838 ***
Vitamin D insuficiency, *n* (%)	103 (57.8%)	15 (60%)	0.93 **
Vitamin D suficiency	75 (42.1%)	10 (40%)	
Leucocytes ×10^3^/µL, median (min.–max.)	6710 (1462–46,100)	11,150 (3240–54,600)	0.0003 ***
Lymphocytes ×10^3^/µL, median (min.–max.)	1270 (282–9967)	1091 (200–7730)	0.099 ***
Neutrophils ×10^3^/µL, median (min.–max.)	5128 9370–36,700)	9506 (1080–51,000)	0.0006 ***
NLR median (min.–max.)	3.75 (0.29–28.9)	6.57 (0.47–72.71)	0.003 ***
PLR median (min.–max.)	170.0 (11.2–683.6)	165.9 (21.7–876.5)	0.376 ***
SII median (min.–max.)	727.4 (36.7–8716.4)	1399 (154.0–9667.8)	0.015 ***
CRP (mg/L), median (min.–max.)	43.1 (0.12–320)	20.9 (0.12–254.0)	0.563 ***
Ferritin (ng/mL), median (min.–max.)	604.5 (90–4555)	950 (243–3647)	0.005 ***
Fibrinogen (mg/dL), median (min.–max.)	455 (122–900)	441 (102–900)	0.863 ***

* Student test, ** chi-square test, *** Mann Whitney test. NLR = neutrophile to lymphocyte ratio; SII = systemic inflammatory index; PLR = platelets to lymphocyte ratio; CRP = C-reactive protein.

**Table 5:** The relationship for the in-hospital mortality with demographic parameters, vitamin D status, and inflammatory biomarkers.

	B	S.E.	Wald	*p*-Value	Exp(B)	95% CI for Exp(B)	
						Lower	Upper
Age	0.039	0.015	5.455	0.015	1.046	1.008	1.094
Sex	0.513	0.406	1.997	0.196	1.572	0.554	3.608
Diabetes mellitus	−0.072	0.662	0.017	0.801	0.822	0.267	3.406
Hypertension	0.257	0.441	0.393	0.575	1.406	0.761	3.041
CCD	0.342	0.458	0.705	0.412	1.363	0.501	3.463
COPD	0.137	1.109	0.014	0.905	1.242	0.152	9.554
NLR	0.106	0.026	12.345	0.0001	1.311	1.052	1.273
PLR	0.002	0.001	4.065	0.034	1.002	1.000	1.005
Ferritin	0.000	0.000	3.100	0.044	1.000	1.000	1.001
CRP	0.001	0.003	0.077	0.751	1.001	0.595	1.007
SII	0.000	0.000	13.090	0.0001	1.000	1.000	1.001
Leucocytes	0.000	0.000	8.423	0.003	1.000	1.000	1.000
Vitamin D	−0.010	0.018	0.180	0.396	0.490	0.955	1.027

CCD = chronic cardiac disease, COPD = chronic obstructive pulmonary disease, NLR = neutrophile to lymphocyte ratio; PLR = platelet to lymphocyte ratio; CRP = C reactive protein; SII = systemic inflammatory index. Multivariate regression of predictive factors used regression of in-hospital mortality as a dependent variable and demographic parameters, vitamin D status, and inflammatory biomarkers as independent predictive variables in a single model. Results are expressed as hazard ratios (Expβ) with 95% CIs.

## Data Availability

The data presented in this study are available on request from the corresponding author.

## References

[1] Bivona G., Agnello L., Ciaccio M. (2018). The immunological implication of the new vitamin D metabolism. Cent. J. Immunol..

[2] Ellison D.L., Moran H.R. (2021). Vitamin D: Vitamin or Hormone?. Nurs. Clin. N. Am..

[3] Demer L.L., Hsu J.J., Tintut Y. (2018). Steroid Hormone Vitamin D. Circ. Res..

[4] Charoenngam N., Holick M.F. (2020). Immunologic Effects of Vitamin D on Human Health and Disease. Nutrients.

[5] Provvedini D.M., Tsoukas C.D., Deftos L.J., Manolagas S.C. (1983). 1,25-dihydroxyvitamin D3 receptors in human leukocytes. Science.

[6] Fiske C.T., Blackman A., Maruri F., Rebeiro P.F., Huaman M., Kator J., Algood H.M.S., Sterling T.R. (2019). Increased vitamin D receptor expression from macrophages after stimulation with M. tuberculosis among persons who have recovered from extrapulmonary tuberculosis. BMC Infect. Dis..

[7] Barragan M., Good M., Kolls J.K. (2015). Regulation of Dendritic Cell Function by Vitamin D. Nutrients.

[8] Kongsbak M., Levring T.B., Geisler C., von Essen M.R. (2013). The vitamin D receptor and T cell function. Front. Immunol..

[9] Chen S., Sims G.P., Chen X.X., Gu Y.Y., Chen S., Lipsky P.E. (2007). Modulatory effects of 1,25-dihydroxyvitamin D3 on human B cell differentiation. J. Immunol..

[10] Carlberg C. (2019). Vitamin D: A Micronutrient Regulating Genes. Curr. Pharm. Des..

[11] Trombetta A.C., Paolino S., Cutolo M. (2018). Vitamin D, Inflammation and Immunity: Review of Literature and Considerations on Recent Translational and Clinical Research Developments. Open Rheumatol. J..

[12] Siddiqui M., Manansala J.S., Abdulrahman H.A., Nasrallah G.K., Smatti M.K., Younes N., Althani A.A., Yassine H.M. (2020). Immune modulatory effects of vitamin d on viral infections. Nutrients.

[13] Lei G.S., Zhang C., Cheng B.H., Lee C.H. (2017). Mechanisms of action of vitamin D as supplemental therapy for Pneumocystis pneumonia. Antimicrob. Agents Chemother..

[14] Ghosh S.K., Weinberg A. (2021). Ramping Up Antimicrobial Peptides against Severe Acute Respiratory Syndrome Coronavirus-2. Front. Mol. Biosci..

[15] Grant W.B., Lahore H., McDonnell S.L., Baggerly C.A., French C.B., Aliano J.L., Bhattoa H.P. (2020). Evidence that vitamin d supplementation could reduce risk of influenza and covid-19 infections and deaths. Nutrients.

[16] Pagnini C., Picchianti-Diamanti A., Bruzzese V., Lorenzetti R., Luchetti M.M., Martin L.S.M., Pica R., Scolieri P., Scribano M.L., Zampaletta C. (2021). Vitamin d signaling in gastro-rheumatology: From immuno-modulation to potential clinical applications. Int. J. Mol. Sci..

[17] Sloka S., Silva C., Wang J., Yong V.W. (2011). Predominance of Th2 polarization by Vitamin D through a STAT6-dependent mechanism. J. Neuroinflammation.

[18] Fisher S.A., Rahimzadeh M., Brierley C., Gration B., Doree C., Kimber C.E., Cajide A.P., Lamikanra A.A., Roberts D.J. (2019). The role of vitamin D in increasing circulating T regulatory cell numbers and modulating T regulatory cell phenotypes in patients with inflammatory disease or in healthy volunteers: A systematic review. PLoS ONE.

[19] Kazemi A., Mohammadi V., Aghababaee S.K., Golzarand M., Clark C.C.T., Babajafari S. (2021). Association of Vitamin D Status with SARS-CoV-2 Infection or COVID-19 Severity: A Systematic Review and Meta-analysis. Adv. Nutr..

[20] Saponaro F., Franzini M., Okoye C., Antognoli R., Campi B., Scalese M., Neri T., Carrozzi L., Monzani F., Zucchi R. (2022). Is There a Crucial Link Between Vitamin D Status and Inflammatory Response in Patients With COVID-19?. Front. Immunol..

[21] Campi I., Gennari L., Merlotti D., Mingiano C., Frosali A., Giovanelli L., Torlasco C., Pengo M.F., Heilbron F., Soranna D. (2021). Vitamin D and COVID-19 severity and related mortality: A prospective study in Italy. BMC Infect. Dis..

[22] Merzon E., Tworowski D., Gorohovski A., Vinker S., Cohen A.G., Green I., Frenkel-Morgenstern M. (2020). Low plasma 25(OH) vitamin D level is associated with increased risk of COVID-19 infection: An Israeli population-based study. FEBS J..

[23] Jevalikar G., Mithal A., Singh A., Sharma R., Farooqui K.J., Mahendru S., Dewan A., Budhiraja S. (2021). Lack of association of baseline 25-hydroxyvitamin D levels with disease severity and mortality in Indian patients hospitalized for COVID-19. Sci. Rep..

[24] Ricci A., Pagliuca A., D’Ascanio M., Innammorato M., De Vitis C., Mancini R., Giovagnoli S., Facchiano F., Sposato B., Anibaldi P. (2021). Circulating Vitamin D levels status and clinical prognostic indices in COVID-19 patients. Respir Res..

[25] Jain A., Chaurasia R., Sengar N.S., Singh M., Mahor S., Narain S. (2020). Analysis of vitamin D level among asymptomatic and critically ill COVID-19 patients and its correlation with inflammatory markers. Sci. Rep..

[26] Hernández J.L., Nan D., Fernandez-Ayala M., García-Unzueta M., Hernández-Hernández M.A., López-Hoyos M., Muñoz-Cacho P., Olmos J.M., Gutiérrez-Cuadra M., Ruiz-Cubillán J.J. (2021). Vitamin D Status in Hospitalized Patients with SARS-CoV-2 Infection. J. Clin. Endocrinol. Metab..

[27] Carpagnano G.E., Di Lecce V., Quaranta V.N., Zito A., Buonamico E., Capozza E., Palumbo A., Di Gioia G., Valerio V.N., Resta O. (2021). Vitamin D deficiency as a predictor of poor prognosis in patients with acute respiratory failure due to COVID-19. J. Endocrinol. Invest..

[28] Chirita-Emandi A., Socolov D., Haivas C., Calapiş A., Gheorghiu C., Puiu M. (2015). Vitamin D Status: A Different Story in the Very Young versus the Very Old Romanian Patients. PLoS ONE.

[29] Niculescu D.A., Capatina C.A.M., Dusceac R., Caragheorgheopol A., Ghemigian A., Poiana C. (2017). Seasonal variation of serum vitamin D levels in Romania. Arch. Osteoporos..

[30] Zugravu C.-A., Soptica F., Tarcea M., Cucu A. (2016). Pertinence of Vitamin D Supplementation In The Adult Romanian Population. Farmacia.

[31] Risk for COVID-19 Infection, Hospitalization, and Death by Age Group|CDC. https://www.cdc.gov/coronavirus/2019-ncov/covid-data/investigations-discovery/hospitalization-death-by-age.html.

[32] WHO—Emergency Situation Reports. https://www.who.int/emergencies/situation-reports.

[33] ROCHE-eLabDoc. https://pim-eservices.roche.com/eLD/web/gb/en/documents.

[34] Holick M.F., Binkley N.C., Bischoff-Ferrari H.A., Gordon C.M., Hanley D.A., Heaney R.P., Murad M.H., Weaver C.M. (2011). Evaluation, treatment, and prevention of vitamin D deficiency: An endocrine society clinical practice guideline. J. Clin. Endocrinol. Metab..

[35] Muhammad S., Fischer I., Naderi S., Jouibari M.F., Abdolreza S., Karimialavijeh E., Aslzadeh S., Mashayekhi M., Zojaji M., Kahlert U.D. (2021). Systemic Inflammatory Index Is a Novel Predictor of Intubation Requirement and Mortality after SARS-CoV-2 Infection. Pathogens.

[36] Dyussenbayev A. (2017). Age Periods of Human Life. Adv. Soc. Sci. Res. J..

[37] Kongsbak-Wismann M., Al-Jaberi F.A.H., Schmidt J.D., Ghanizada M., Hansen C.B., Lopez D.V., Woetmann A., Ødum N., Bonefeld C.M., Stryhn A. (2021). Normal T and B Cell Responses Against SARS-CoV-2 in a Family With a Non-Functional Vitamin D Receptor: A Case Report. Front. Immunol..

[38] Dror A.A., Morozov N., Daoud A., Namir Y., Yakir O., Shachar Y., Lifshitz M., Segal E., Fisher L., Mizrachi M. (2022). Pre-infection 25-hydroxyvitamin D3 levels and association with severity of COVID-19 illness. PLoS ONE.

[39] Gallelli L., Chiara Mannino G., Luciani F., de Sire A., Mancuso E., Gangemi P., Cosco L., Monea G., Averta C., Minchella P. (2021). Vitamin D Serum Levels in Subjects Tested for SARS-CoV-2: What Are the Differences among Acute, Healed, and Negative COVID-19 Patients? A Multicenter Real-Practice Study Vitamin D Serum Levels in Subjects. Nutrients.

[40] De Smet D., De Smet K., Herroelen P., Gryspeerdt S., Martens G.A. (2021). Serum 25(OH)D Level on Hospital Admission Associated With COVID-19 Stage and Mortality. Am. J. Clin. Pathol..

[41] White J.H. (2022). Emerging Roles of Vitamin D-Induced Antimicrobial Peptides in Antiviral Innate Immunity. Nutrients.

[42] Bishop E., Ismailova A., Dimeloe S., Hewison M., White J.H. (2021). Vitamin D and Immune Regulation: Antibacterial, Antiviral, Anti-Inflammatory. JBMR Plus.

[43] Fabri M., Stenger S., Shin D.M., Yuk J.M., Liu P.T., Realegeno S., Lee H.M., Krutzik S.R., Schenk M., Sieling P.A. (2011). Vitamin D Is Required for IFN-γ–Mediated Antimicrobial Activity of Human Macrophages. Sci. Transl. Med..

[44] Coveney C., Tellier M., Lu F., Maleki-Toyserkani S., Jones R., Bart V.M.T., Jones R., Coveney C., Lu F., Tellier M. (2020). Innate immunology in COVID-19—A living review. Part I: Viral entry, sensing and evasion. Oxf. Open Immunol..

[45] Rodrigues P.R.S., Alrubayyi A., Pring E., Bart V.M.T., Jones R., Coveney C., Lu F., Tellier M., Maleki-Toyserkani S., Richter F.C. (2020). Innate immunology in COVID-19—A living review. Part II: Dysregulated inflammation drives immunopathology. Oxf. Open Immunol..

[46] Murai I.H., Fernandes A.L., Sales L.P., Pinto A.J., Goessler K.F., Duran C.S.C., Silva C.B., Franco A.S., Macedo M.B., Dalmolin H.H. (2021). Effect of a Single High Dose of Vitamin D3 on Hospital Length of Stay in Patients With Moderate to Severe COVID-19: A Randomized Clinical Trial. JAMA.

[47] Mardani R., Alamdary A., Mousavi Nasab S.D., Gholami R., Ahmadi N., Gholami A. (2020). Association of vitamin D with the modulation of the disease severity in COVID-19. Virus Res..

[48] Sergeev I.N. (2021). Vitamin D and COVID-19: How much vitamin d does a man need?. Nutrients.

[49] Alkhafaji D., Al Argan R., Albaker W., Al Elq A., Al-Hariri M., Alsaid A., Alwaheed A., Alqatari S., Alzaki A., Alwarthan S. (2022). The Impact of Vitamin D Level on the Severity and Outcome of Hospitalized Patients with COVID-19 Disease. Int. J. Gen. Med..

[50] Davoudi A., Najafi N., Aarabi M., Tayebi A., Nikaeen R., Izadyar H., Salar Z., Delavarian L., Vaseghi N., Daftarian Z. (2021). Lack of association between vitamin D insufficiency and clinical outcomes of patients with COVID-19 infection. BMC Infect. Dis..

[51] Butler-Laporte G., Nakanishi T., Mooser V., Morrison D.R., Abdullah T., Adeleye O., Mamlouk N., Kimchi N., Afrasiabi Z., Rezk N. (2021). Vitamin D and COVID-19 susceptibility and severity in the COVID-19 host genetics initiative: A Mendelian randomization study. PLoS Med..

[52] Hastie C.E., Pell J.P., Sattar N. (2021). Vitamin D and COVID-19 infection and mortality in UK Biobank. Eur. J. Nutr..

[53] Sun Y., Zhou J., Ye K. (2021). Extensive Mendelian randomization study identifies potential causal risk factors for severe COVID-19. Commun. Med..

[54] Liu D., Tian Q.Y., Zhang J., Hou H.F., Li Y., Wang W., Meng Q., Wang Y.X. (2021). Association between 25 Hydroxyvitamin D Concentrations and the Risk of COVID-19: A mendelian randomization study. Biomed. Environ. Sci..

